# Modulation of Huh7.5 Spheroid Formation and Functionality Using Modified PEG-Based Hydrogels of Different Stiffness

**DOI:** 10.1371/journal.pone.0118123

**Published:** 2015-02-18

**Authors:** Bae Hoon Lee, Myung Hee Kim, Jae Ho Lee, Dror Seliktar, Nam-Joon Cho, Lay Poh Tan

**Affiliations:** 1 School of Materials Science and Engineering, Nanyang Technological University, Singapore, Singapore; 2 Department of Biomedical Engineering, Technion—Israel Institute of Technology, Haifa, Israel; North Carolina A&T State University, UNITED STATES

## Abstract

Physical cues, such as cell microenvironment stiffness, are known to be important factors in modulating cellular behaviors such as differentiation, viability, and proliferation. Apart from being able to trigger these effects, mechanical stiffness tuning is a very convenient approach that could be implemented readily into smart scaffold designs. In this study, fibrinogen-modified poly(ethylene glycol)-diacrylate (PEG-DA) based hydrogels with tunable mechanical properties were synthesized and applied to control the spheroid formation and liver-like function of encapsulated Huh7.5 cells in an engineered, three-dimensional liver tissue model. By controlling hydrogel stiffness (0.1–6 kPa) as a cue for mechanotransduction representing different stiffness of a normal liver and a diseased cirrhotic liver, spheroids ranging from 50 to 200 μm were formed over a three week time-span. Hydrogels with better compliance (i.e. lower stiffness) promoted formation of larger spheroids. The highest rates of cell proliferation, albumin secretion, and CYP450 expression were all observed for spheroids in less stiff hydrogels like a normal liver in a healthy state. We also identified that the hydrogel modification by incorporation of PEGylated-fibrinogen within the hydrogel matrix enhanced cell survival and functionality possibly owing to more binding of autocrine fibronectin. Taken together, our findings establish guidelines to control the formation of Huh7.5 cell spheroids in modified PEGDA based hydrogels. These spheroids may serve as models for applications such as screening of pharmacological drug candidates.

## Introduction

The liver is a soft tissue with around 2 kPa shear storage modulus in a healthy state [1,2] and is composed of about 80% hepatocytes and 20% non-parenchymal cells such as stellate cells, sinusoidal endothelial cells, and Kupffer cells.[3] Hepatocytes in the liver exist in a polygonal shape and are highly polarized to show at least two apical surfaces and two basolateral surfaces.[3] Isolation of primary hepatocytes can be important for cell-based treatment and drug screening purposes. However, when isolated hepatocytes are cultured in two-dimensional (2D) culture using highly stiff tissue culture flasks with about 1 GPa elastic modulus, they lose their native morphology and functionality, which subsequently hinders their effectiveness in applications such as toxicity screening of drug metabolites.[4] Development of a culture system mimicking the *in vivo* structure and functions of the liver cells still remains a challenge.

Three-dimensional (3D) cultures for primary hepatocytes proved to be better in maintaining hepatocyte phenotype and cell polarization. It has been reported that culturing hepatocytes between collagen layers (the so-called ‘sandwich culture’) could be a better alternative for primary hepatocyte culture.[5,6] Sandwich systems can help to promote a polygonal morphology of primary hepatocytes and retain functionality of hepatocytes compared to a monolayer hepatocyte culture on polystyrene tissue culture flask. This is attributed to cell-matrix interaction from the top and bottom collagen layers as well as the good cell-cell binding between neighboring hepatocytes as compared to a flattened morphology in the monolayer culture of hepatocytes.

Another promising approach is the 3D spheroid model which can mimic closely cell behaviors akin to physiological architectures compared to 2D tissue culture in terms of cell morphology and cell functionality. As these hepatocyte spheroids display polarized cell morphology and direct cell-cell contacts just like *in vivo* systems, they are good models for drug metabolism and toxicology studies.[7,8] Studies have shown that cells of spheroids grown in 3D scaffolds such as Matrigel and PureMatrix exhibited a better hepatic functionality than those in 2D like sandwich cultures.[9] Spheroid culture has been performed with various methods such as agitation culture, spontaneous self-assembly in non-adhesive wells, and 3D scaffolds.[10,11,12,13] Spheroids can be prepared by encapsulating hepatocytes or immortalized hepatocellular carcinoma (HCC)-derived cells such as Huh7 and HepG2 in several hydrogels or cell-compatible matrices such as PEG-based hydrogels, alginate hydrogels, Matrigel, and galactosylated cellulosic sponge.[13,14,15,16,17,18,19] Spheroids obtained from immortal human hepatocytes are as useful as primary hepatocytes for drug toxicity test.[20] The structure of the hydrogels could be an important cue for spheroid formation as well as its functionality.[21,22] The structural features of the hydrogels could be manipulated by changing matrix chemistry, stiffness, and porosity.[4,14]

PEG is a bio-inert, non-toxic material with mechanical properties and functionalities that could be easily tailored via changing molecular weights and modifying end groups with bioactive molecules.[23,24] PEG-based hydrogels have been extensively used for cell encapsulation.[25,26,27] Also, hepatocytes and hepatocellular carcinoma (HCC)-derived cells such as Huh7.5 and HepG2 have been shown to form spheroids inside PEG-based hydrogels and exhibited hepatocyte function to a certain level;[28,29,30,31,32] even encapsulated immortal human hepatocytes inside PEG-based hydrogels showed their capability to be infected by viruses, indicating that a PEG-based hydrogel platform could be useful for hepatitis virus studies.[30,31] Underhill et al. reported that degree of cell-cell interactions, the presence of adhesive sequences (RGDs), and PEG chain length could affect hepatocellular function inside 3D PEG-based hydrogels.[33] Williams et al. proved that PEG-fibrinogen gels could provide a good environment for 3 D spheroid culture, making primary hepatocytes in them retain autocrine growth factors (epidermal growth factor, hepatocyte growth factor, and transforming growth factor-α) and autocrine fibronectin, which could maintain hepatocyte functionality.[29] However, effect of stiffness of PEG-based hydrogels mimicking a heathy liver and a fibrotic liver on spheroid formation and its functionality of hepatic cell lines has not been systematically explored. Physical properties of the cellular microenvironment have been known to have significant effects on cellular behaviors.[34,35] Stiffness for example has been shown to effectively modulate cellular differentiation into multiple lineages[36,37,38] as well as tumor migration.[39]

In the present study, we hypothesized that spheroid formation and functionality inside PEG-based hydrogels could be modulated dominantly by hydrogel stiffness as a cue for mechanotransduction. To this end, a human hepatocellular carcinoma cell line (Huh7.5) was chosen as a model cell since Huh7.5 cells have been used for liver function study and for virus infection study at the initial stage.[30,31] These Huh7.5 cells were encapsulated into PEG-based hydrogels with 0.1–6 kPa complex modulus, covering a range of a normal liver storage shear modulus of around 2 kPa to a diseased liver shear modulus of around 5 kPa.[40] Spheroid formation and functionality inside PEG-based hydrogels were investigated in detail through microscopy (inverted microscopy and immunofluorescence microscopy), SEM (scanning electron microscope), and ELISA (*enzyme-linked immunosorbant* assay).

## Materials and Methods

### Sample Preparation

Poly(ethylene glycol)-diol (PEG-OH) with a 10-kDa molecular weight (Aldrich) was dehydrated by azeotropic distillation of water in toluene and was vacuum-dried at around 90°C. Acrylation of linear PEG-OH was conducted under argon by reacting with distilled acryloyl chloride (Sigma-Aldrich) and distilled triethylamine (Aldrich) in anhydrous dichloromethane (Aldrich) solution of PEG-OH at an acryloyl chloride: OH molar ratio of 2:1. The resulting PEG-diacrylate (PEGDA) was precipitated in anhydrous diethyl ether and dried under vacuum at room temperature for 2 days. For further purification, PEGDA was dialysed against distilled water using a dialysis membrane (MWCO = 3.5 kDa) at 4°C for 1 day and was lyophilized to obtain a purified PEGDA.[41] Proton NMR (^1^H-NMR) was used to validate the expected product formation (above 95% acrylation).

PEGDA was conjugated to fibrinogen to make PEGDA-fibrinogen (PF) hydrogel precursor solution.[23,41] In brief, Bovine fibrinogen (Sigma-Aldrich) was dissolved in 150 mM phosphate buffered saline (PBS) containing 8 M urea to a final concentration of 7 mg/ml. Tris (2-carboxyethyl) phosphine hydrochloride (TCEP, Sigma) was added to the fibrinogen solution at a molar ratio of 1.5 (TCEP):1 (fibrinogen thiols) to reduce disulfides in fibrinogen. After dissolution, the pH of the solution was adjusted to 8 using 5 M NaOH solution. A solution of PEGDA (280 mg/ml) in 150 mM PBS with 8 M urea was added in a molar excess of four times over the fibrinogen thiols and allowed to react for 3–5 h at room temperature in the dark. After the reaction was completed, the reaction solution was diluted with 150 mM PBS containing 8 M urea in 1:1 ratio, and precipitated by adding four volumes of acetone at room temperature in a separation funnel. The precipitate was re-dissolved, homogenized, and dialyzed against 150 mM PBS at 4°C for 48 h with four changes of PBS in dialysis tubing (MWCO = 50 kDa).

### Cell-seeded PEG-based Constructs

The Huh7.5 human hepatocarcinoma cell line was purchased from RIKEN (VA, Japan). The Huh7.5 cells were maintained in Dulbecco’s modified eagle medium (DMEM; 10569010 Invitrogen Life technique, NY, US) supplemented with 10% foetal bovine serum (SV30160.03IR, Hyclone, Thermo Fisher scientific, US) and 1% penicillin/streptomycin (10378016; Invitrogen life technique, NY, USA) at a temperature of 37°C in a humidified atmosphere containing 5% CO_2_.

**Fig 1:**
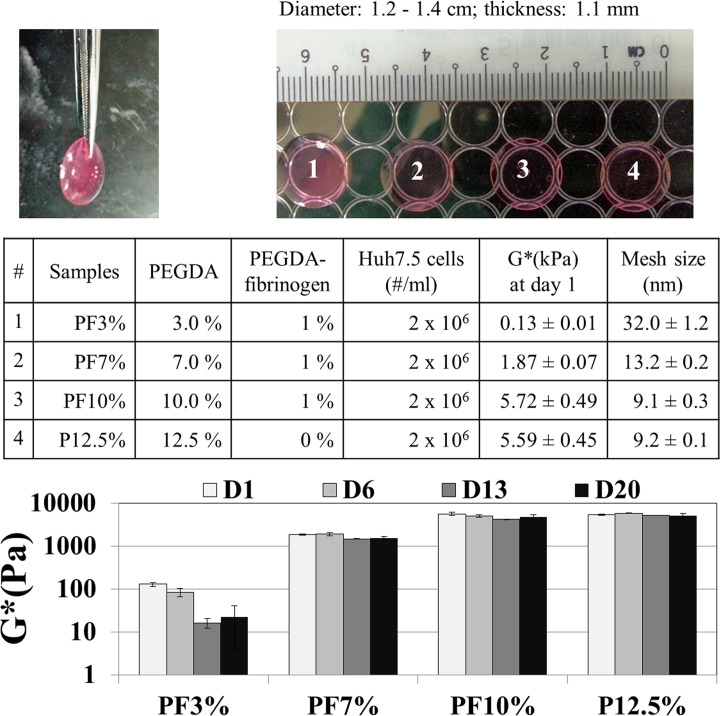
Huh7.5 cell-laden hydrogels with different stiffness (0.1–6 kPa) as a cue for mechanotransduction representing various stiffness of a normal liver and a diseased cirrhotic liver. All the Huh7.5 cell-laden hydrogels exhibited viscoelastic properties and varying concentrations of PEGDA (3–12.5%) were used to control stiffness of the hydrogel matrix, as determined by rheological measurements. The stiffness of the hydrogels increased in the order of PF3% (3% PEGDA + 1% PF, 0.13 kPa ± 0.01) < PF7% (7% PEGDA + 1% PF, 1.87 kPa±0.07) < PF10% (10% PEGDA +1% PF, 5.72 kPa ± 0.49) ≅ P12.5% (12.5% PEGDA, 5.59 kPa ± 0.45). A higher addition of PEGDA exhibited a higher complex modulus of the Huh7.5 cell-laden hydrogels (one way ANOVA, p < 0.001, n = 4, among PF3%, PF7% and PF10%). The initial complex shear modulus of PF10% (5.72 kPa ± 0.49) and P12.5% (5.59 kPa ± 0.45) were not statistically different (P > 0.05).

Cell-seeded PEG-based hydrogels were prepared by crosslinking the PEGDA solution (3,7, and 10% w/v) containing PEGDA-fibrinogen (10 mg/ml) according to published protocols[23]. In brief, cell-seeded constructs were made from 50 μl aliquots of the cells in a suspension of PEG-based solutions combined with 0.1% w/v Irgacure 2959 photoinitiator solution (Ciba Specialty Chemicals) (final cell density = 2×10^6^ cells/ml) using a flat bottom 96-well plate cover as the moulding template as seen in Fig. 1. After photopolymerization by 365 nm UV lamp irradiation for 3 min, the cell-seeded hydrogels were transferred into 12 well plates with cell culture medium (2 ml). Encapsulation efficiency was above 90%. PEGDA hydrogel constructs containing PEGDA-fibrinogen were compared to a control group, PEGDA hydrogel only. For spheroid formation of the seeded Huh7.5 cells, all constructs were monitored for cell viability using Live/Dead solution containing 2μM calcein AM and 4 mM ethidium homodimer-I stains for 60 min at 37°C. The labelled cells were digitally imaged. Huh 7.5 proliferation was also tested using the cell proliferation reagent WST-8 (2-(2-methoxy-4-nitrophenyl)-3-(4-nitrophenyl)-5-(2,4-disulfophenyl)-2H-tetrazolium) during the incubation of 20 days. Briefly, 10% WST-8 reagent was added to each well of 12 well tissue culture polystyrene (TCPS) containing cell-laden PF hydrogel with 600 μl culture media and the plates were incubated for 4 h at 37°C. The absorbance at 450 nm of water soluble formazan dye solution in the culture medium was measured.

### PEG-based Hydrogels with Different Mechanical Properties

The viscoelastic properties of cell-laden hydrogels were characterized using steady shear and sinusoidal shear rheometry. Frequency-sweep measurements were performed using Anton Paar Physica MCR 501 equipped with a Peltier plate temperature-controlled base and the 25 mm cone-plate geometry with a cone angle of 1 degree or parallel plate with 10 mm diameter. The testing conditions for all measurements were 2% strain amplitude at an oscillation frequency of 0.1–10 Hz within the *linear viscoelastic regime*. The complex shear modulus is calculated by G* = [(G’)^2^+(G”)^2^]^1/2^, G’ = shear storage modulus and G” = shear loss modulus.

### Immunofluorescence Imaging with Confocal Microscopy

We investigated cell proliferation and liver cell metabolic activity of spheroids inside PEG-based hydrogels by Ki-67, fibronectin, albumin, CYP 450, and occludin antibodies. Spheroids in each hydrogel sample were fixed in 4% paraformaldehyde for 30 min in room temperature. Following fixation, constructs were washed 3 x with phosphate buffered saline (PBS), permeabilized with 0.1% Triton X-100, blocked using 2% bovine serum albumin (BSA) in PBS for 1 h in 4°C, and then stained by mouse anti-Ki-67 monoclonal antibody FITC (*Life Technologies*) overnight at 4°C. After washing 3 x at room temperature, F-actin and cell nuclei were stained with Alexa Fluor *555 phalloidin (Life Technologies)* and DAPI (4',6-diamidino-2-phenylindole, Life Technologies), respectively. Immunofluorescence labelling of each hydrogel sample was performed by applying primary antibodies against albumin (monoclonal mouse anti-human (F-10: SC-271605)), fibronectin (polyclonal rabbit anti-human (H-300: SC-9068)), cytochrome 450 (monoclonal mouse anti-human (SC-53850)), and tight junction (occludin: monoclonal mouse anti-human (F-7: SC-271842)) at 1:50 dilution in PBS/BSA buffer at 4°C overnight. After washing 3 x 10 min in PBS/BSA buffer, secondary antibodies (AF 488 goat anti-mouse, AF 488 goat anti-rabbit, AF 488 anti-goat, and AF 555 rabbit anti-mouse Invitrogen) were applied at 1:200 dilution in PBS/BSA buffer at room temperature for 60 min. Hydrogel constructs were then imaged via confocal microscopy (Leica TCS SP8 and LSM700 META (Carl Zeiss AG, Oberkochen, Germany)).

### Western Blot

We performed western blot analysis with a liver activity marker such as albumin and a scaffolding marker of liver tissue formation as occludin. PEG-based hydrogels containing Huh7.5 cell spheroids were collected and washed by PBS. Each sample was re-suspended in lysis buffer (iNtRON biotech, Korea) and sonicated for 10 min on ice. Afterwards, the completely lysed samples were centrifuged at 15000 rpm for 30 min. Supernatant was collected and stored at -80°C until running SDS-PAGE. Before running, the samples were boiled with 4x laemmli sample buffer (161–0747: Bio-Rad) for 5 min. A volume of twenty microliters of each boiled sample was loaded to wells of an 8% SDS-PAGE gel, and the proteins were trans-blotted to a nitrocellulose membrane (162–0232: Bio-Rad) at 350 mA for about 2 h. The trans-blotted membrane was blocked by 5% fat free milk TBST (pH7.6 Tris buffer with 0.05% Tween-20) for 1 h at room temperature. Primary antibodies (mouse anti-albumin (F-10: SC-271605), occludin (F-7: SC-271842), and mouse anti-beta-actin (SC-47778) antibodies; Santa Cruz Biotechnology, Santa Cruz, CA) were diluted at 1:1,000 in the 2% fat free milk TBST solution and incubated with the membrane overnight in a cooled chamber. The blotted membrane was washed by TBST and incubated with horseradish peroxidase-conjugated anti-mouse secondary IgG. Immunoreactive bands were detected using ECL kit (170–5060: Bio-Rad). The band image and intensity was analyzed using an Amersham Imager 600 apparatus (GE Healthcare, Buckinghamshire, UK).

### Albumin Secretion

Culture medium samples were collected at a designated time and stored at -20°C. Albumin concentration was determined using an enzyme-linked immunosorbent assay, Albumin human ELISA kit (Abcam). The assay was performed according to the manufacturer’s instructions. Albumin secretion from each hydrogel with Huh7.5 cells (1x10^5^ cells) initially loaded was measured and compared. All the data for the parameters were normalized to the amount of albumin on day 1.

### Morphology

A scanning electron microscope (SEM) was used to investigate the spheroid morphology. Cross-sections of lyophilized PEG-based hydrogel samples were mounted and sputter-coated with gold. They were examined under SEM (JEOL 6360) at an accelerating voltage of 5 kV and working distance of 13 mm.

### Statistical Analysis

Statistical analysis was performed using the Microsoft Excel statistical analysis software package. Comparisons between multiple treatments (samples 1, 2, and 3) were made with analysis of variance (ANOVA) and comparisons between two treatments (samples 3 and 4) were made using a two-tail student’s *t*-test. Standard deviation was calculated and presented for each treatment group (mean ± SD). The level of significance was set to p < 0.05.

## Results

### Properties of PEG-based Hydrogels

Cell-laden PEG-based hydrogels were prepared by crosslinking PEGDA polymer chains containing the PEGDA fibrinogen (PF) precursor using photo-polymerization in the presence of a Huh 7.5 suspension (2x10^6^ cells / ml) as seen in Fig. 1. All the Huh7.5 cell-laden hydrogels exhibited viscoelastic properties and varying concentrations of PEGDA (3–12.5%) were used to control stiffness of the Huh7.5 cell laden hydrogels, as determined by rheological measurements. A higher addition of PEGDA exhibited a higher complex modulus of the Huh7.5 cell-laden hydrogels (one way ANOVA, *p* < 0.001, *n* = 4 measurements of two samples). The PEGDA-fibrinogen hydrogel (PF3%) with the lowest PEGDA concentration at 3% exhibited a complex modulus of 0.13 kPa ± 0.01, followed by PF7% (7% PEGDA + 1% PF) at 1.87 kPa ± 0.07, and PF10% (10% PEGDA + 1% PF) at 5.72 kPa ± 0.49. The complex modulus of unmodified PEGDA (P12.5%) at 5.59 kPa ± 0.45 is similar to that of PF10%. During 20 days in cell culture, the mechanical stiffness of Huh7.5 cell-laden hydrogels did not decrease much except for PF3% (3% PEGDA + 1% PF), which could be more labile to remodelling of matrix after spheroid growth. This is possibly owing to the higher ratio of fibrinogen to PEGDA. The compliance (the inverse of stiffness) of cell-laden hydrogels increased in the opposite trend of the complex modulus. Huh7.5 cell-laden hydrogels with complex shear moduli ranging from 0.1 kPa to 6 kPa were subsequently used for the proceeding study on spheroid formation and functionality. Also, the average mesh size (distance from two entanglement points) of the hydrogels was calculated from the rubber elasticity theory using the equation, ${\xi =\left(\frac{G^{'}N_{a}}{RT}\right)}^{{-}_{3}^{1}}$, where G’ is the storage modulus, N_a_ is the Avogadro constant, R is the molar gas constant, and T is the temperature.[42] All the hydrogels exhibited mesh sizes below 50 nm. It is expected that PF3% with the mesh size of 32 nm ± 1.2 may have a bigger porosity than PF10% with the mesh size of 9.1 nm ± 0.3.

### Spheroid Formation in 3D PEG- based Hydrogels

**Fig 2:**
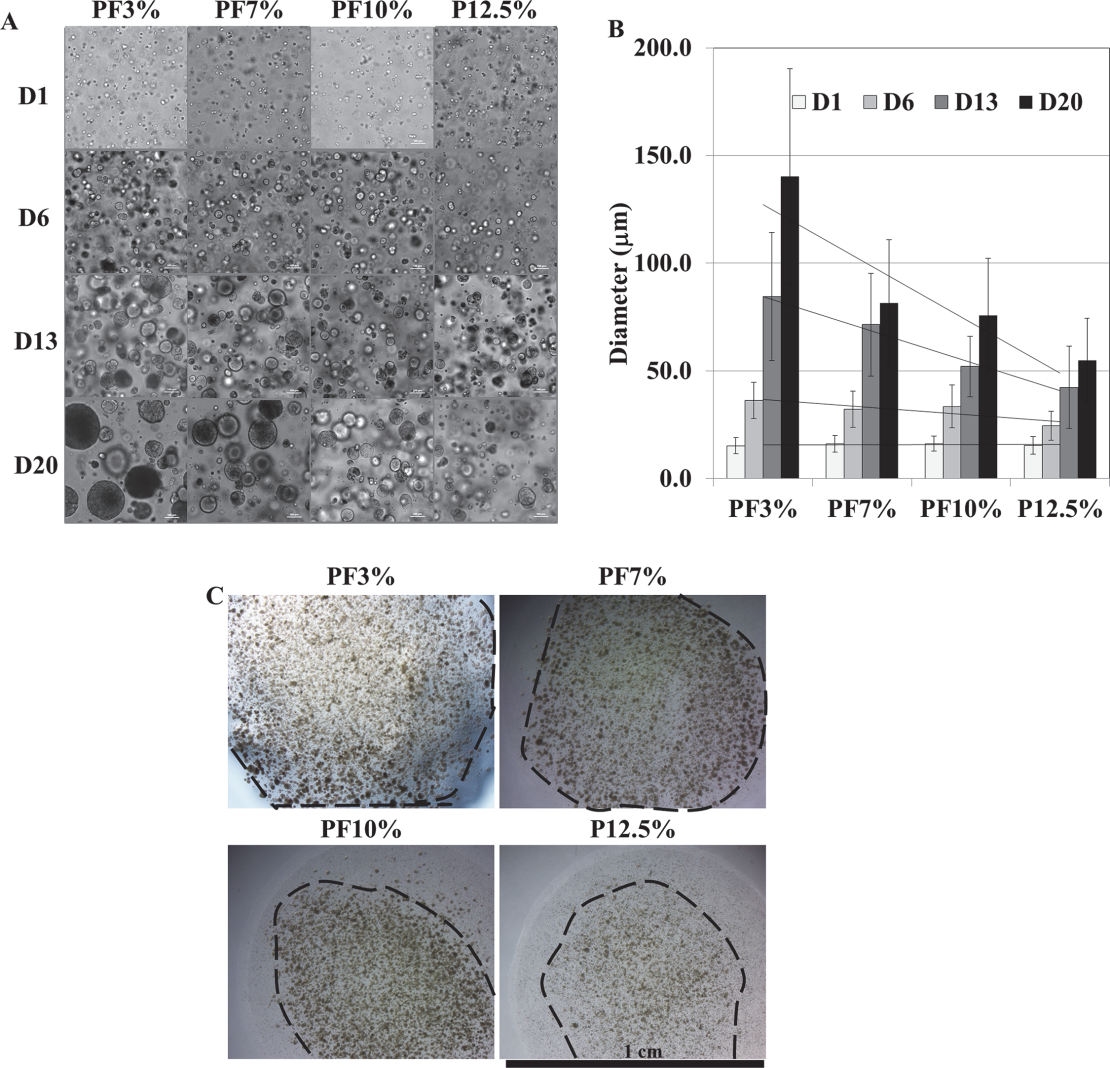
Time dependent spheroid growth in Huh7.5 cell-laden hydrogels for 20 days. A. The overall morphology of the Huh7.5 cells within the PEG-based hydrogels was monitored and observed over a period of 20 days using contrast-enhanced light microscope. Scale bar = 100 μm. The Huh7.5cells inside all PEG-based hydrogels aggregated to form spheroids over time. B. The size of spheroids inside PEG-based hydrogels grew bigger at different rates in all samples. One-way ANOVA for PF3%, PF7%, and PF10% was performed revealing significant differences between the samples after day 1 (P > 0.5, P < 0.05, P < 0.001, and P < 0.001 at days 1, 6, 13, and 20, respectively); spheroid sizes of PF10% and P12.5% were statistically different (P < 0.01) at days 6, 13, and 20. C. The area occupied by spheroids recognizable by the naked eye inside PEG-based hydrogels increased in the order of P12.5% < PF10% < PF7% < PF3%. Scale bar = 1 cm.

**Fig 3:**
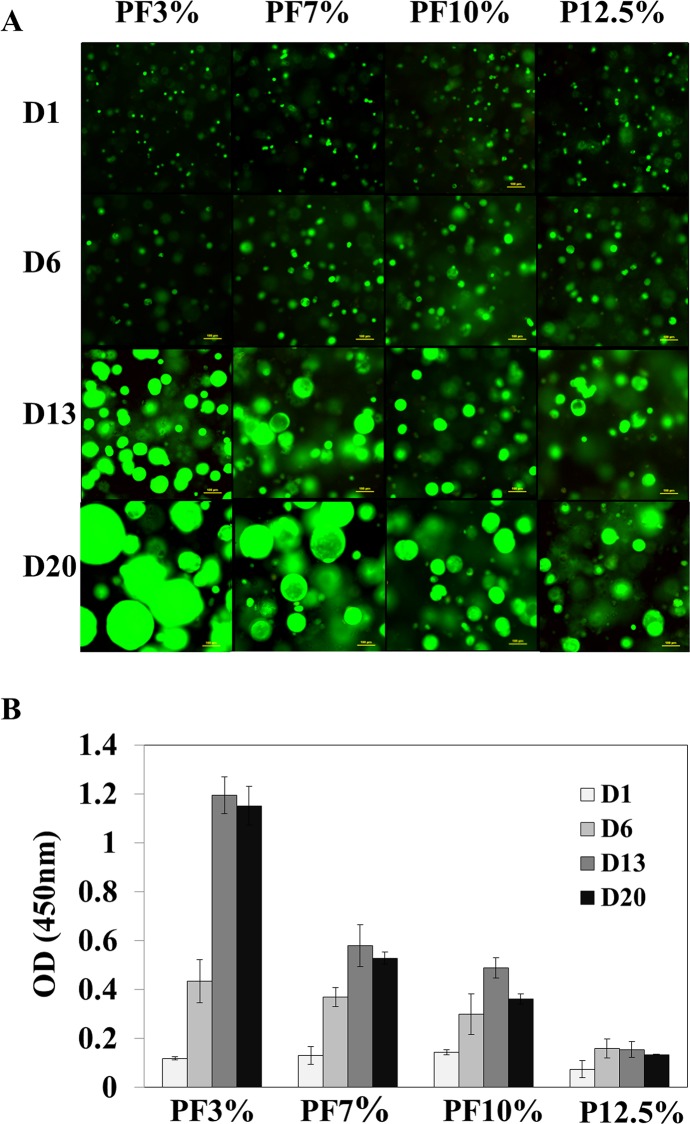
Cell viability test of Huh7.5 cell-laden hydrogels. A. live dead images of spheroids inside PEG-based hydrogels. Most Huh7.5 cells insides PEG-based hydrogels survived and formed spheroids with different sizes over a period of 20 days. Scale bar = 100 μm. B. Cell viability and cell proliferation reagent WST-8 measurement (One way ANOVA, n = 3: P < 0.0001 at day 13 and day 20 among PF3%, PF7%, and PF10%; t-test (n = 3, PF10% and P12.5%): P < 0.01 at day 13 and day 20). The results exhibited a trend of increased proliferation until 13 days in most of PEG-based hydrogels.

The overall morphology of the Huh7.5 cells within the PEG-based hydrogels was monitored and observed over a period of 20 days using contrast-enhanced light microscope. The Huh7.5 cells inside all PEG-based hydrogels aggregated to form spheroids over time as seen in Figs. 2 and 3. The size of spheroids inside PEG-based hydrogels became bigger with time in all samples. There was a quantitative difference observed and measured by image J in the size of the spheroids (n = 68) in the various hydrogel compositions tested. As seen in Fig. 2, for PF3% (3% PEGDA + 1% PF), the size of spheroids increased from 15.2 μm ± 3.7 at day 1 to 36.3 μm ± 8.3 at day 6, 84.5 μm ± 29.7 at day 13, and 140.1 μm ± 50.2 at day 20; for PF7% (7% PEGDA + 1% PF), the size of spheroids increased from 16.1 μm ± 3.8 at day 1 to 32.3 μm ± 8.4 at day 6, 71.5 μm ± 23.9 at day 13, and 81.4 μm ± 29.4 at day 20; for PF10% (10% PEGDA + 1% PF), the size of spheroids increased from 16.3 μm ± 3.5 at day 1 to 33.5 μm ± 10.0 at day 6, 52.1 μm ± 14.1 at day 13, and 75.8 μm ± 26.5 at day 20; for P12.5% (12.5% PEGDA), the size of spheroids increased from 15.5 μm ± 4.2 at day 1 to 24.5 μm ± 6.6 at day 6, 42.4 μm ± 19.0 at day 13, and 55.0 μm ± 19.35 at day 20. One-way ANOVA for samples 1, 2, and 3 was performed, revealing significant differences between the treatments after day 1 (P > 0.5, P < 0.05, P < 0.001, and P < 0.001 at day 1, day 6, day 13, and day 20, respectively). Overall, PF3% and PF7% formed much bigger spheroids than PF10% and P12.5%. Spheroid sizes of PF10% and P12.5% were statistically different (P < 0.01) at days 6, 13, and 20. The area occupied by spheroids inside PEG-based hydrogels increased in the order of P12.5% < PF10% < PF7% < PF3%. The spheroids at the edge of all hydrogels appeared to grow bigger compared to spheroids in the core probably owing to more available oxygen and nutrients. PF7% displayed a more homogenous spheroid population inside the hydrogel compared to PF3%. Cell viability and proliferation within 3D PEG-based hydrogels were characterized spectrophotometrically using a quantitative cell viability and cell proliferation reagent (WST-8). The proliferation results shown in Fig. 3 demonstrate a trend of increased proliferation until 13 days in most of PEG-based hydrogels. Even though there was no significant difference among samples at day 1 and day 6, PF3% and PF7% exhibited higher cell viability than PF10% and P12.5%. At day 13 and day 20, the more compliant PEG-based hydrogels showed a higher viability (one way ANOVA, n = 3: P < 0.0001 at day 13 and day 20 among samples 1, 2, and 3). When comparing PF10% and P12.5%, which are of similar stiffness, PF10% had a significantly higher value compared to P12.5% (P < 0.01 at day 13 and day 20; t-test, n = 3)

**Fig 4:**
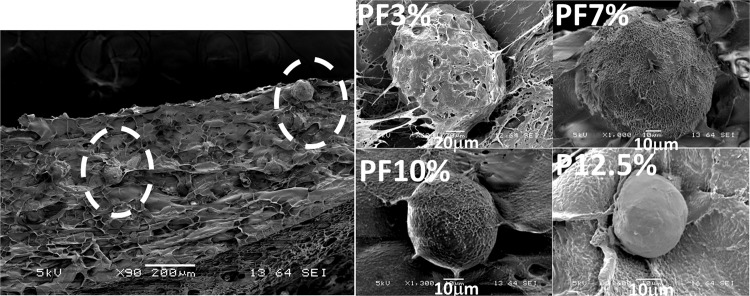
Scanning electron microscope (SEM) pictures of spheroids on cross-sectioned samples. The spheroids of stiffer matrices (PF10% and P12.5%) appeared smaller and more compact than those of PF3% and PF7%. Spheroids of more compliant matrices (PF3% and PF7%) displayed rougher surfaces than those of PF10% and P12.5%. Especially, the spheroid of the most compliant matrix (PF3%) seemed to show micro-pores on the surface, suggesting that there could be some channel-like structures in the spheroid.

We sought to identify the spheroid formations in the ultrastructure of the lyophilized PEG-based Huh7.5 cell-laden hydrogels using scanning electron microscope (SEM) as seen in Fig. 4. Spheroids of Huh7.5 cells in lyophilized hydrogels were observed within a cross-section of SEM samples; the spheroids of stiffer matrices (PF10% and P12.5%) appeared smaller and more compact than those of PF3% and PF7%. Spheroids of less stiff matrices (PF3% and PF7%) displayed rougher surfaces than those of PF10% and P12.5%. Especially, the spheroid of the most compliant matrix, PF3%, seemed to show micro-pores on the surface, suggesting that there could be some channels in the spheroid.[17]

### Spheroid Functionality in 3D PEG-based Hydrogels

**Fig 5:**
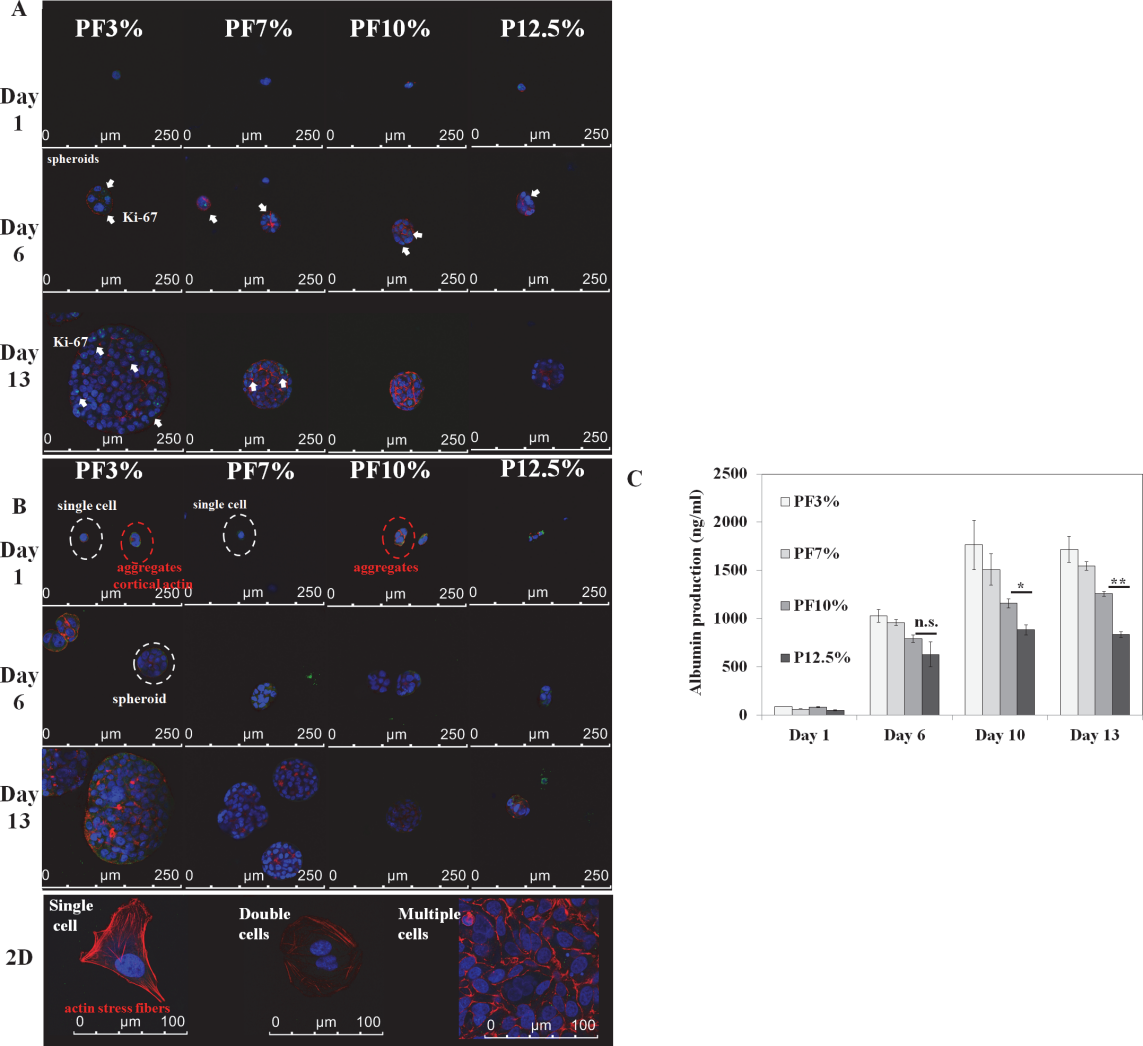
Time dependent spheroid formation and albumin expression inside Huh7.5 cell-laden hydrogels at day 1, day 6, and day 13. Spheroid growth was observed in Huh7.5 cell-laden hydrogels through F-actin, nucleus proliferation marker (Ki-67), and albumin staining. A. blue: nucleus; red: F-actin; green: Ki-67. B. blue: nucleus; red: F-actin; green: albumin. After spheroids were formed, cortical type red F-actin could be observed among Huh7.5 cells. On the contrary, Huh7.5 cells cultured on 2D glass slide displayed well developed actin stress fibers in single and double cells. C. ELISA for quantification of albumin secretion from each hydrogel with Huh7.5 cells (1x10^5^ cells) initially loaded was performed. One way ANOVA, n = 3: P < 0.01, P < 0.05 and P < 0.001 at day 6, day 10, and day 13, respectively among PF3%, PF7%, and PF10%; t-test (n = 3 between PF10% and P12.5%): *: P < 0.05 at day 10; **: P < 0.001 at day 13; n.s.: not significant.

**Fig 6:**
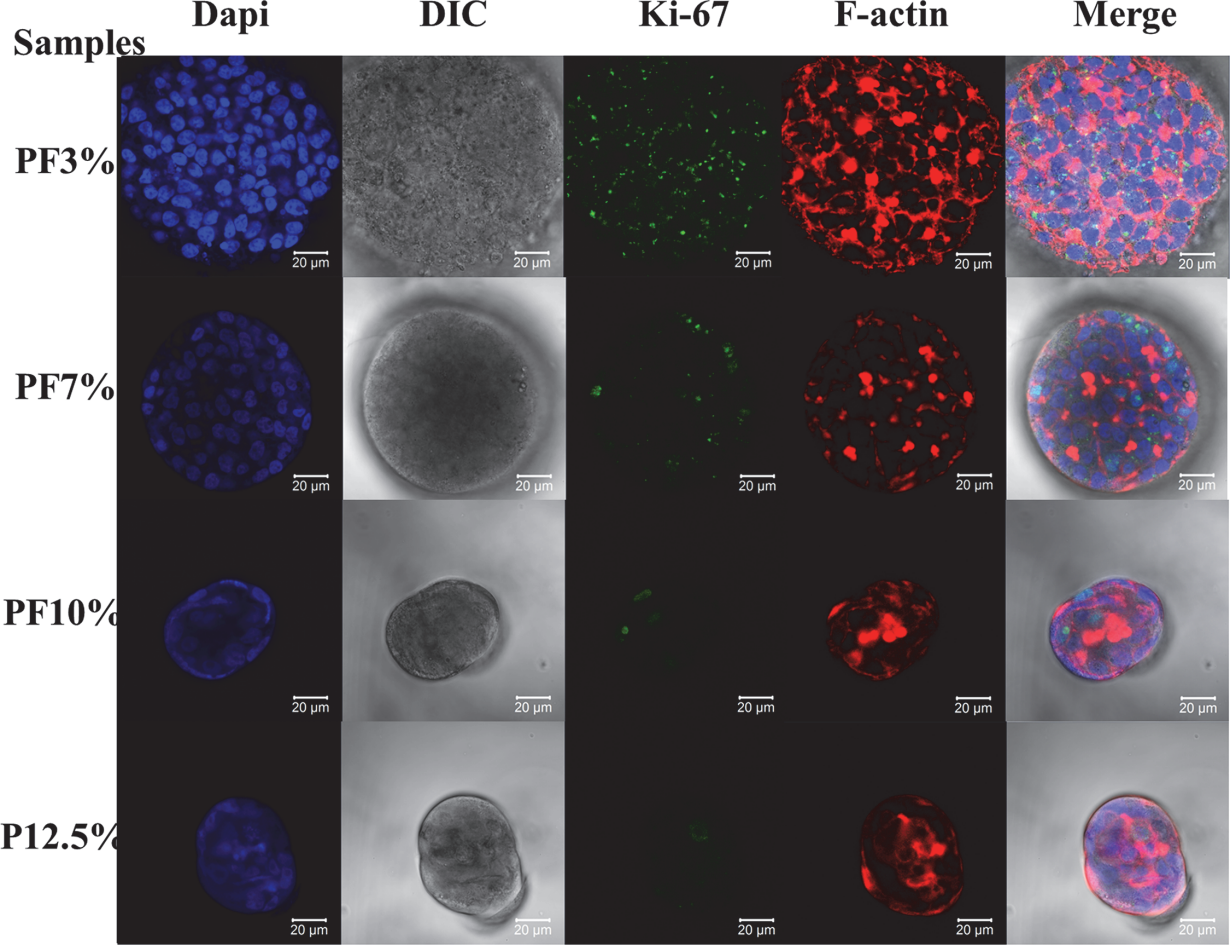
Ki-67 nucleus protein staining of spheroids inside Huh7.5 cell-laden hydrogels at day 20. Blue: nucleus; green: Ki-67 (a proliferation protein marker); red: F-actin. Spheroids in the most compliant hydrogel PF3% displayed well distributed dot-like Ki -67 expression even in the center. Spheroids in PF7% and PF10% had some Ki-67 expression, predominantly on the edge. P12.5% seemed to have minimal expression of Ki -67 protein.

**Fig 7:**
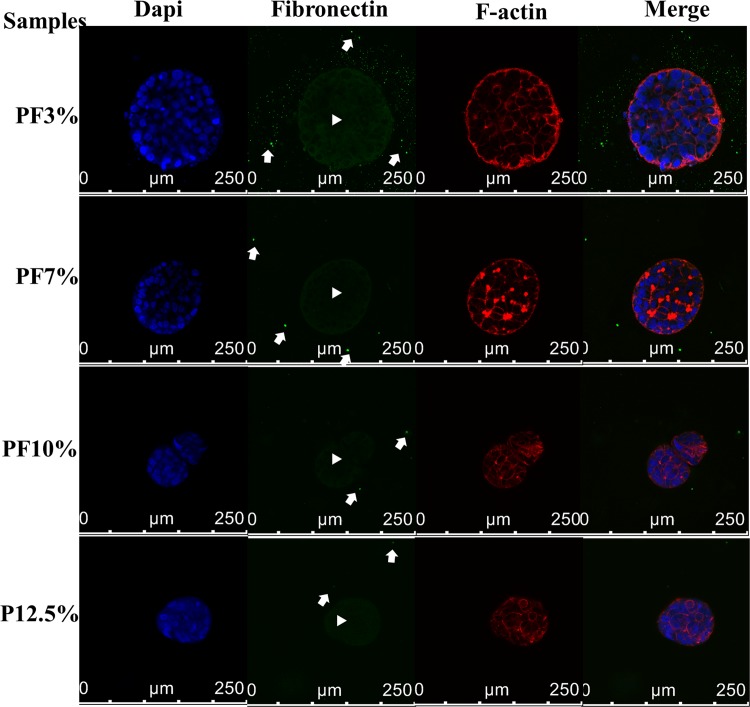
Autocrine fibronectin staining of spheroids inside Huh7.5 cell-laden hydrogels at day 20. White arrow head: fibronectin captured inside spheroids; white arrow: fibronectin crystalized outside spheroids. Autocrine fibronectin was observed inside all samples. Modified PEG-based hydrogels containing PEGDA-fibrinogen (PF) seemed to produce and hold more fibronectin inside and outside spheroids than PEGDA only hydrogels (P12.5%). The fibrinogen moiety in modified PEG-based hydrogels indirectly may facilitate incorporation of autocrine fibronectin.

**Fig 8:**
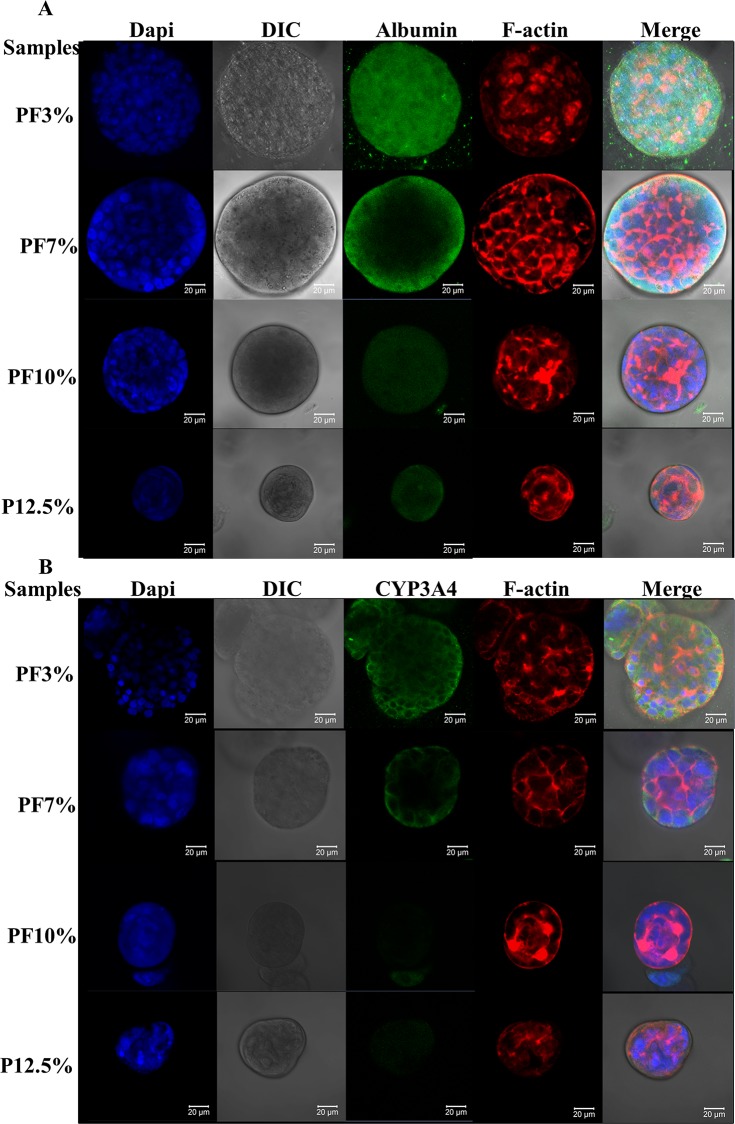
Albumin and CYP 3A4 staining of spheroids inside Huh7.5 cell laden hydrogels at day 20. A. blue: nucleus; green: albumin; red: F-actin. B. blue: nucleus; green: CYP 3A4; red: F-actin. Spheroids in the most compliant hydrogel (PF3%) displayed the highest protein expression of both albumin and CYP 3A4 (cytochrome P450 3A4, an enzyme of liver). The extent of CYP 3A4 expression decreased markedly from the compliant to stiff samples, especially in P12.5%, indicating that Huh7.5 cells in the compact spheroids of P12.5% might not function well.

Huh7.5 cell spheroids initially began as a single cell encapsulated inside the cell-laden hydrogels at day 1 and started to aggregate to form a spheroid through cell migration or proliferation with time as seen in Fig. 5. Proliferation could become a more dominant factor for spheroid growth in more compliant hydrogels (PF3% and PF7%). After spheroids were formed, cortical type red F-actin could be observed among Huh7.5 cells. On the contrary, Huh7.5 cells cultured on 2D glass slide displayed well developed actin stress fibers in a single cell and double cells. As the spheroids grew larger, albumin expression also appeared more obvious. In order to delineate spheroid functionality in the PEG-based hydrogels, we evaluated Ki-67 nucleus protein expression as a proliferation marker, albumin protein expression and CYP 450 enzyme expression as metabolic activity markers of spheroids in 3D PEG-based hydrogels. Towards this end, spheroids within each hydrogel sample were fixed after 20 day and stained for F-actin (red), cell nuclei (blue), and proteins (Ki-67, fibronectin, albumin, and CYP 3A4; green), and imaged at a cross section near the centre of each spheroid via confocal microscopy. Spheroids within all samples displayed polarized assembly of F-actin between Huh7.5 cells as seen in Figs. 6, 7, and 8. In terms of Ki-67 proliferation protein marker expression in Huh7.5 cell nucleus, spheroids in the most compliant hydrogel, PF3%, displayed well distributed dot-like Ki-67 expression even in the centre. Spheroids in PF7% and PF10% had some Ki-67 expression predominantly on the edge. P12.5% seemed to have minimal expression of Ki-67 protein. Fibronectin produced by Huh7.5 cells was observed in cytoplasm inside spheroids of all samples. Some fibronectin was found outside spheroids of PF3%, PF7%, and PF10%. When it comes to albumin expression and CYP 3A4 enzyme activity, we observed that spheroids in the most compliant hydrogel, PF3%, displayed the highest protein expression of both albumin and CYP 3A4. The extent of CYP 3A4 expression decreased markedly from the compliant to stiff samples, especially in sample 4 (P12.5%), indicating that Huh7.5 cells in compact spheroids of sample 4 might not function well.

**Fig 9:**
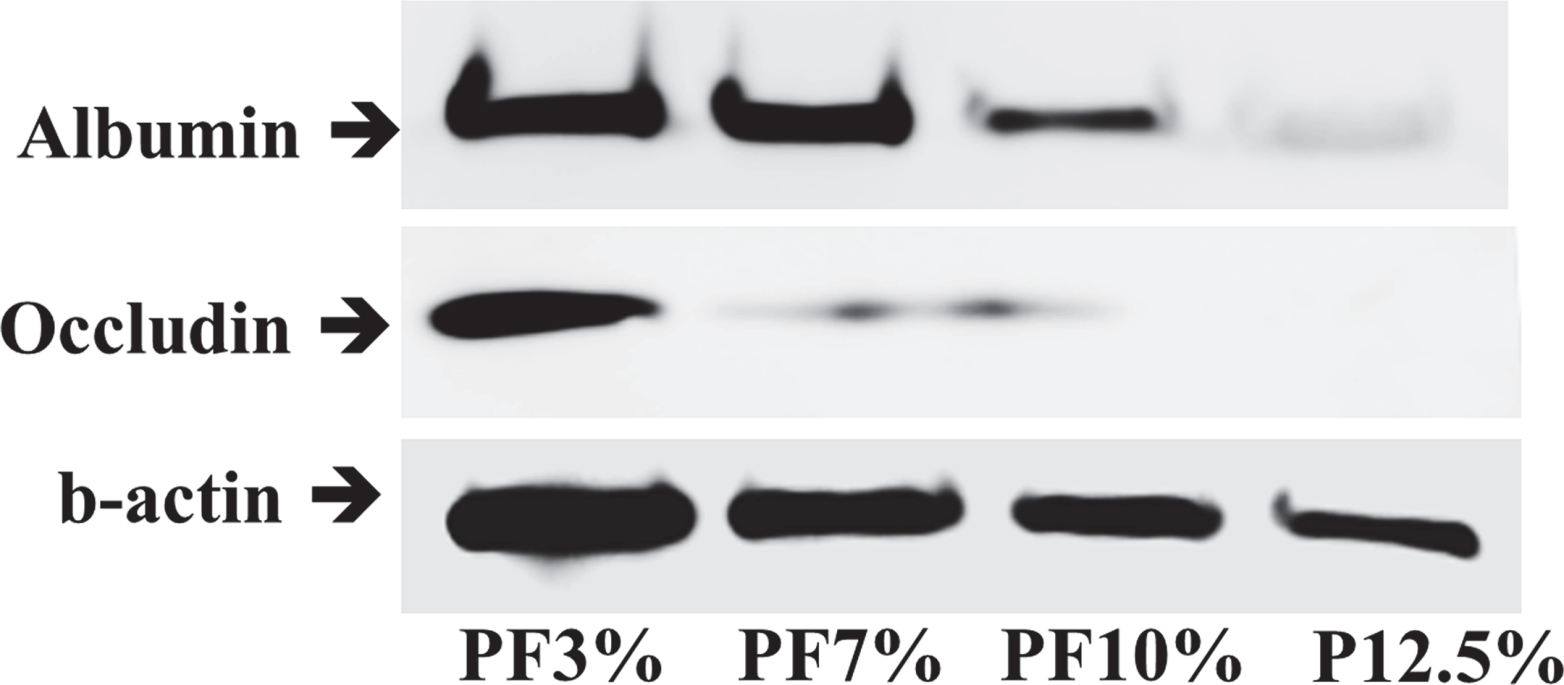
The levels of albumin and tight junction occludin protein were analyzed by western blot at day 20. Expression of occludin and albumin from all hydrogel samples was observed. Overall, level of albumin from spheroids inside PEG-based hydrogels increased in the order of P12.5% (12.5% PEGDA) < PF10% (10% PEGDA + 1% PF) < PF7% (7% PEGDA + 1% PF) < PF3% (3% PEGDA + 1% PF). Spheroids in PF3% and PF7% produced higher albumin at day 20 compared to PF 10% and P12.5%. The highest level of occludin was expressed in the most compliant gel (PF3%).

**Fig 10:**
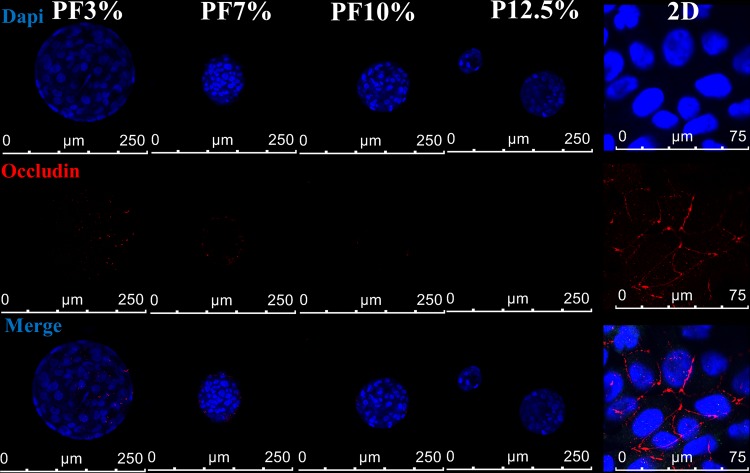
Occludin expression inside Huh7.5 cell laden hydrogels (PF3%, PF7%, PF10%, and P12.5%) at day 20 (Blue: nucleus; red: occludin). Immunofluorescence labelling of each hydrogel sample was performed by applying primary occludin antibody (monoclonal mouse anti-human), secondary antibody (AF 555 rabbit anti-mouse), and DAPI (4',6-diamidino-2-phenylindole). Occludin was localized along the multiple apical domains in spheroids of the more compliant hydrogels (PF3% and PF7%), which is similar to in vivo occludin localization. Occludin was expressed between Huh7.5 cells on 2D glass, indicating that Huh 7.5 cells in 2D culture showed a simple epithelial cell polarity.

Fig. 9 showed albumin and occludin levels per each hydrogel by means of western blot. Expression of albumin from all samples was observed. Overall, level of albumin from spheroids inside PEG-based hydrogels increased in the order of P12.5% (12.5% PEGDA) < PF10% (10% PEGDA + 1% PF) < PF7% (7% PEGDA + 1% PF) ≤ PF3% (3% PEGDA + 1% PF). Spheroids in the more compliant hydrogels (PF3% and PF7%) produced the higher albumin at day 20 compared to PF10% and P12.5%, which is consistent with albumin immunochemistry results in Fig. 8. Occludin (a 65 kDa integral plasma membrane tight junction protein) was expressed in PF3%, PF7%, and PF10%. Spheroids in the most compliant hydrogel, PF3%, clearly expressed the most amount of occludin tight junction, which meant that spheroids inside the hydrogel had good cell-cell contacts. This phenomenon was reconfirmed by immunofluorescence confocal microscopy of occludin tight junction as seen in Fig. 10. In the 2D culture, occludin was localized between cells, indicating that Huh 7.5 cells in the 2D culture showed a normal epithelial cell polarity. Spheroids of Huh7.5 cells in 3D hydrogels expressed occludin tight junction marker in a different manner compared to the 2D culture. Occludin was clearly localized along the multiple apical domain in spheroids of the more compliant hydrogels (PF3% and PF7%), which is similar to cellular localizations in vivo.[17] Overall, our results reveal that the compliant hydrogels mimicking a soft healthy liver can effectively promote albumin production and CYP3A4 activity.

## Discussion

Modifying the network structure of the cell-laden PEG-based hydrogels by varying PEGDA concentrations to alter their stiffness can affect Huh7.5 cell spheroid viability and growth rate.[43] The physical cues afforded by the encapsulating hydrogel microenvironment including matrix stiffness may be important factors in determining size and functionality of the Huh7.5 spheroids in 3D. In 2D cultures, hepatocytes and hepatocellular carcinoma cell lines (HepG2 and Huh7.5 cells) are known to be sensitive and respond to the mechanical properties of their substrates. Hepatocytes seeded on soft Matrigel (34 Pa) quickly formed spheroids, whereas on stiffer materials or 2D, hepatocytes exhibited polygonal morphology and did not aggregate effectively.[21,32,44] Generally, encapsulated cells inside nonadhesive soft materials such as agarose and unmodified PEG hydrogels aggregated into multicellular spheroids over time.[45,46] In our PEG-based hydrogel system (nonadhesive PEGDA plus PEGDA fibrinogen or nonadhesive PEGDA only), Huh7.5 cells in the 3D PEG-based hydrogels aggregated and formed multicellular spheroids with 50~200 μm diameter within 20 days. Though it may sound difficult that a single cell with initial 10 μm diameter can migrate and build up cell-cell contacts inside PEG-based hydrogels with much smaller theoretical mesh sizes below a few hundreds Å,[23,30] our work shows that the encapsulated Huh7.5 cells inside PEG-based hydrogels managed to aggregate and to form a spheroid depending on the stiffness of the hydrogels. It has been reported that viral particles with 100 nm diameter can penetrate PEG-based hydrogels to infect the encapsulated Huh 7.5 cells, indicating that there could be micro-fractures and defects inside cell-laden hydrogels owing to the presence of cells during photopolymerization.[30,31] Not only do the Huh7.5 cells form spheroids; it is evident from our study that the spheroid formation is highly modulated by the cross-link density of the PEG-based hydrogels, which can affect cell migration and cell proliferation in defected cell-laden hydrogels. The expression results of Ki-67 nucleus protein (cell proliferation marker) showed that Huh7.5 cells inside PEG-based hydrogels could proliferate, which promoted spheroid growth especially in the compliant hydrogels. The more compliant hydrogels with complex shear modulus of 2 kPa and below (PF3% and PF7%) supported better spheroid formation and spheroid growth compared to stiffer PEG-based hydrogels (PF10% and P12.5%), indicating that the compliant gels could support Huh7.5 cell migration and cell growth, leading to multicellular spheroid growth.

It has been reported that hepatoma multicellular spheroid formation goes through three stages: 1) aggregate formation of dispersed cells via integrin (β1)-ECM (extracellular matrices: collagen, fibronectin, and laminin); 2) a delay phase of E-cadherin expression and accumulation; 3) formation of strong cell-cell contacts via homophilic cadherin-cadherin binding.[46,47,48,49] Even though fibrinogen moiety is present in modified PEG-based hydrogels containing PEGDA, the fibrinogen may not directly bind to integrin (α_5_β_1_) on hepatocytes. It may indirectly interact with hepatocytes through hepatocyte secreted autocrine fibronectin which can bind to fibrin.[29,50,51] We found that autocrine fibronectin was observed inside all spheroids; however modified PEG-based hydrogels containing PEGDA-fibrinogen (PF) seemed to produce and hold more fibronectin inside and outside spheroids than PEGDA hydrogels only (P12.5%), which could promote cell-matrix interaction and cell (spheroid) growth. Therefore it is believed that the fibrinogen moiety incorporated in our PEG-based hydrogel indirectly facilitated the building of strong cell-cell contacts that is required for spheroid formation via promotion of autocrine fibronectin secretion.[29]

Hepatocyte spheroids can exhibit direct cell-cell interaction, cell polarity, and an improved liver specific functionality.[52] The actin cytoskeleton in hepatocyte spheroids does not form stress fibers but localizes on the cortex.[12] Similarly, in our system, the Huh7.5 cell spheroids in modified PEG-based hydrogels exhibited a cortical actin web instead of stress fibers as seen in early 2D culture. Huh7.5 cell spheroids can help to maintain differentiation and functionality of Huh7.5 cells, resulting from their good cellular contacts and polarized structures. In our study, the Huh7.5 cell spheroids in PEG-based hydrogels exhibited good functionality in terms of albumin production and CYP 3A4 expression, and these expressions were observed to be dependent on physical stiffness of the PEG-based hydrogels. Huh7.5 cell spheroids in more compliant PEG-based hydrogels (PF3% and PF7%) exhibited a better albumin production and CYP 3A4 expression compared to stiffer hydrogels (PF10% and P12.5%). A more compliant matrix could be more favourable for diffusion of oxygen and nutrients. In the liver, polarity of hepatocytes is important to maintain liver function. Tight junctional proteins such as ZO-1 and occludin may have an important role for Huh7.5 polarity as well as hepatocyte polarity. In the 2D culture, most occludin was expressed between Huh7.5 cells, meaning that Huh7.5 cells in the 2D culture exhibited an epithelial cell polarity unlike in 3D. Spheroids of Huh7.5 cells in 3D hydrogels expressed occludin tight junction marker in a different mode compared to the 2D culture. Occludin was localized along the multiple apical domain in spheroids of the more compliant hydrogels (PF3% and PF7%), which closely resembles cellular localizations of occludin in vivo. Overall, Huh 7.5 cells in spheroids of PEG-based hydrogels exhibited improved tight junctional localization and expressed albumin and occludin protein significantly, indicating that 3D is superior than 2D in regulating the functionality of Huh7.5 cells.

Albumin production and cell viability of Huh7.5 cell spheroids inside PEG-based hydrogels reached the maximum within two weeks and were maintained thereafter. The control of spheroid diameter is essential to sustain high Huh7.5 functionality, because if the spheroids get too large, cells in the core go through oxygen depletion and necrosis.[53] Spheroids of around 150–300μm usually show a proliferative zone at the surface, quiescent zones in the middle and a hypoxic zone (necrotic zone) in the core.[54] When the diameter of Huh7.5 cell spheroids grew bigger than 200 μm over 20 days, the functionality of spheroids did not improve further from day 13 where the spheroid size had probably reached the maximum for optimum exchange and diffusion of gases and nutrients. Though necrotic foci were not observed, Huh7.5 cells in spheroids could be expected to go through necrosis in the centre zone.

In conclusion, we report on Huh7.5 cell spheroid formation and functionality in PEG-based hydrogels, and their response to matrix stiffness in the range of 0.1–6 kPa, mimicking both a normal compliant liver and a diseased stiff liver. Spheroid formation was observed in all the modified as well as unmodified PEG-based hydrogels. However, the sizes of spheroids were significantly affected by several factors, including the modulus and component of hydrogels, as well as the time in culture. Hydrogel modulus seemed to be the dominant factor in controlling spheroid formation and its functionality. The more compliant hydrogels with up to 2 kPa shear modulus supported bigger spheroids in a shorter time and better cell viability and functionality. We can conclude that the hydrogels with up to 2 kPa shear modulus could represent health liver tissues which are soft and flexible to effectively regenerate themselves. When comparing the PEG-based hydrogel modified with fibrinogen with the unmodified PEGDA gel, the former supported bigger spheroid formation and better functionality. Our systematic study on control over spheroid formation and size through mechanical stiffness of modified PEG-base hydrogels reveals important insight about mechanobiology of healthy liver cells and tissues. This knowledge could also serve as a useful platform for further development of controlled 3D spheroid co-culture with non-parenchymal cells for studies in drug toxicology screening or virus-infection.
